# Microstructure and properties of TC4 titanium alloy micro-arc oxide composite coating based on laser surface texturing

**DOI:** 10.1038/s41598-025-89056-3

**Published:** 2025-03-20

**Authors:** Xin Ma, Qiang Shi, Yong Huang, Yaqi Liu, Xu Yue

**Affiliations:** 1 College of Mechanical and Electrical Engineering, Xinjiang Institute of Engineering, Urumqi, 830023 China; 2 College of Control Engineering, Xinjiang Institute of Engineering, Urumqi, 830023 China; 3 Xinjiang Xiangrun New Materials Technology Co., Ltd, Hami, 839000 China

**Keywords:** Titanium alloys, Laser surface texturing, Micro-arc oxidation, Microstructure, Properties, Composites, Ceramics, Metals and alloys

## Abstract

Micro-arc oxidation technology is one of the important means to improve the tribological properties of titanium alloys, but the bonding performance between the micro-arc oxidation film and the substrate material limits the further application of the micro-arc oxidation film. This study investigates TC4 titanium alloy as a model system, the microstructure and properties of the composite coating were obtained by laser surface texturing composite micro-arc oxidation process. The microstructure, phase composition, and worn morphology of the Micro-arc oxidation coating were characterized using X-ray diffraction, field emission scanning electron microscopy, and optical microscopy, respectively. Mechanical properties, including hardness, friction coefficient, and adhesion strength, were assessed using microhardness tester, Bruker tribometer, and micro/nano scratch tester, respectively. Results revealed that the Micro-arc oxidation coatings predominantly consist of Rutile TiO₂ and Anatase TiO₂ phases. The coating thickness in the mixed electrolyte reached up to 23.82 μm, with a maximum hardness of 467.56 HV₀.₅. Additionally, the friction coefficient of the laser-textured Micro-arc oxidation coatings decreased to 0.3. Laser surface texturing significantly enhanced the adhesion strength between the coating and substrate while simultaneously improving the tribological performance.

## Introduction

Titanium alloys are hindered by inherent disadvantages such as low surface hardness, high friction coefficients, and poor wear resistance, significantly restricting their applications, particularly in tribology^1,2^. Enhancing the friction and wear resistance of titanium alloys is therefore critical for broadening their applicability. Compared with traditional surface modification technologies (such as plasma spraying^3,4^, thermal spraying^5^, etc.), micro-arc oxidation technology can greatly improve the wear resistance of titanium alloys. Micro-arc oxidation (MAO), also referred to as plasma electrolytic oxidation (PEO), is an emerging surface modification technology that enables the in-situ formation of ceramic oxide coatings on metallic substrates^6^. This process relies on localised, instantaneous high-temperature and high-pressure sintering, achieved through anodic oxidation in an electrolyte under applied voltage, which induces uniform sub-discharges across the electrode surface^7^. By employing MAO, the surface properties, composition, and structure of materials can be fundamentally transformed, making it one of the most effective approaches for improving the tribological performance of titanium alloy^8^. However, the differences in chemical bonding characteristics between the micro-arc oxidation coating and the substrate, as well as the large surface porosity caused by the chemical oxidation of MAO, can weaken the bonding strength between the oxide coating and the substrate^9^.

Conventional approaches, such as optimizing electrolyte composition, prolonging the discharge duration, or increasing the applied voltage or current, can moderately enhance the bonding strength of MAO coatings. However, these methods often lead to excessive ion release, which significantly alters the local pH of the electrolyte near the substrate surface, thereby compromising the corrosion resistance of the coating^10,11^. Numerous studies have demonstrated that integrating techniques like mechanical milling^12^, sputtering^13,14^, hot-dipping^15^, and multi-arc ion plating^16^with MAO can modify the surface structure of alloys, yielding improvements in the frictional and wear performance of composite coatings. Among various techniques, laser surface texturing (LST) has demonstrated the ability to enhance the interfacial bonding between the coating and substrate. By creating microtextured arrays with precise geometric dimensions and arrangements, this method increases surface roughness of the substrate, reduces disparities in chemical bonding properties, and provides a higher specific surface area and surface free energy, thereby improving the adhesion strength of the coating to the substrate^17,18^. LST offers additional advantages, including operational simplicity, high processing precision, environmental friendliness, and excellent repeatability, making it a highly promising technology. Saetang^19^reported that TiN-coated AISI M2 high-speed steel with laser-textured surfaces exhibited reduced friction and wear under a 50 N load. Similarly, LST has been shown to enhance the joint shear strength of Al/CFRP hybrid joints in friction stir welding^20^. Wang et al.^21^ successfully joined dissimilar W-Cu butt joints, achieving tensile strengths up to 201 MPa and shear strengths up to 140 MPa through LST. These findings highlight the potential of LST to significantly enhance interfacial bonding between coatings and substrates.

In this paper, two types of LST designed on the surface of TC4 titanium alloy. The laser-textured TC4 substrates were subjected to MAO in a mixed electrolyte, and the resulting composite coating were analyzed for their phase composition, microstructure, hardness, adhesion strength, and tribological properties. The findings offer an experimental foundation for the engineering application of high-bond-strength MAO coating, contributing to the broader utilization of titanium alloys in advanced applications.

## Materials and methods

### Materials

TC4 titanium alloy (Ti-6Al-4 V) was selected as the experimental substrate, with its chemical composition detailed in Table 1.

**Table 1:** The chemical composition of TC4 substrate (wt/%).

Elements	Al	V	Fe	C	*N*	H	O	Ti
Content	6.2	4.1	0.3	0.08	0.05	0.015	0.2	Bal.

### Methodology

**Fig. 1:**
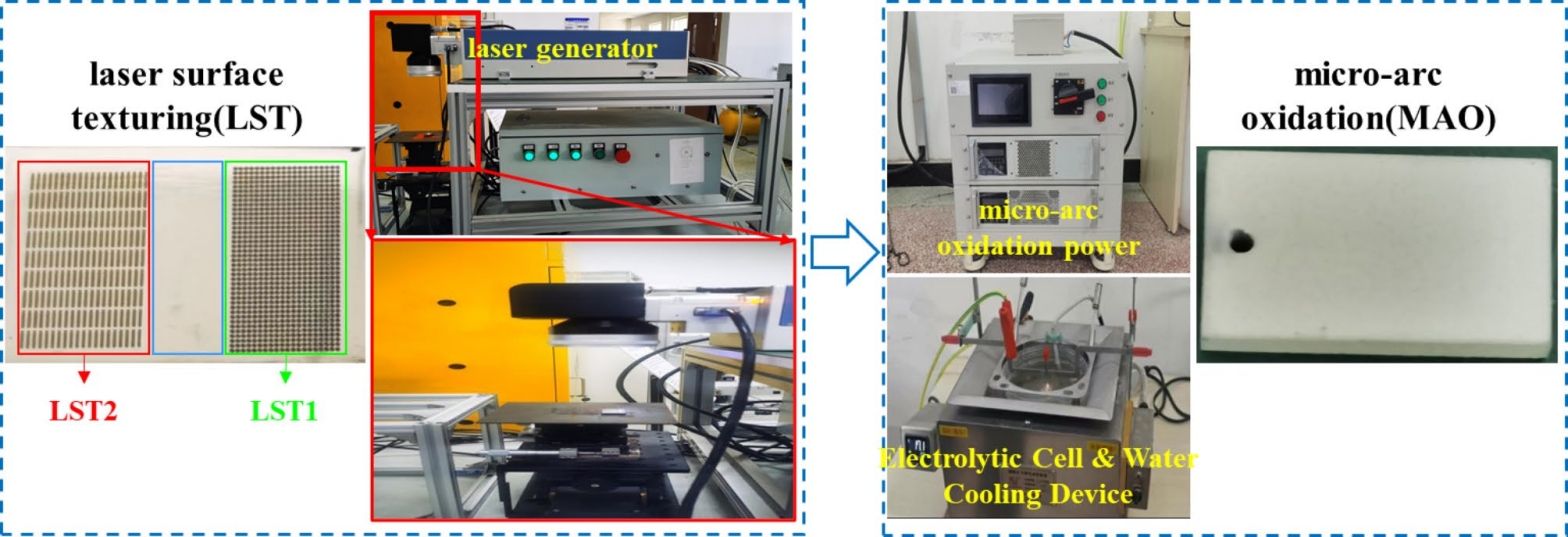
The process for the Laser textured MAO composite coating.

The TC4 substrate was cut into dimensions of 30 mm × 20 mm × 5 mm by wire cutting. The process for preparing the composite coating on the TC4 surface is illustrated in Fig. 1, which comprises LST and MAO. Initially, the TC4 substrate surface was sequentially polished with 600#, 1000#, 1500#, and 2000# sandpapers, followed by ultrasonic cleaning in acetone and deionized water for 5 min each, and air-dried to ensure the removal of contaminants and oxidation coating. Subsequently, a 355 nm UV laser was employed to texture the pre-treated TC4 specimens. The laser processing parameters included a pulse frequency of 50 kHz, a pulse width of 1 µs, a current of 1 A, and a scanning speed of 500 mm/s. Two types of LST patterns were fabricated: a circular ring texture (LST1) and a rectangular ring texture (LST2). The LST1 featured 150 μm in diameter, 14 μm in depth, and 100 μm in spacing, while the LST2 exhibited 100 μm in width, 12 μm in depth, 200 μm in spacing, and 1,500 μm in length. The MAO was conducted using a constant-current power supply with a current density of 6 A/dm², a voltage of 650 V, a frequency of 800 Hz, and a duty cycle of 25%. The mixed electrolyte consisted of 10 g/L sodium silicate (Na₂SiO₃·9 H₂O), 5 g/L trisodium phosphate (Na₃PO₄·12 H₂O), 5 g/L sodium hexametaphosphate ((NaPO₃)₆), and 2 g/L sodium hydroxide (NaOH). The pH of the weakly alkaline electrolyte was maintained between 8 and 13, and the electrolyte temperature was controlled at 30 ± 5 °C for 30 min. After LST and MAO, the TC4 specimens were ultrasonically cleaned in deionized water and anhydrous ethanol, followed by air drying.

The physical phase composite of the coating was analyzed using a Rigaku Smart Lab 9 kW X-ray polycrystalline diffractometer (XRD) with a scanning speed of 2°/min and a range of 20°–80°. A Gemini SEM 500 field emission scanning electron microscope (SEM) was employed to examine the surface topography, cross-sectional morphology, and worn morphology of the MAO coating, while the elemental composition and distribution were determined using energy-dispersive spectroscopy (EDS). The pore size of the coating was measured using ImageJ image analysis software. The coating thickness was evaluated at 500× magnification using an FCM5000W metallurgical microscope, and the average value was obtained from 10 measurements. Hardness measurements of the MAO coating was conducted using an HVS-1000 A digital microhardness tester with a 500 g load and a loading duration of 10 s. Friction performance of the coating was assessed using a UMT Tribolab Bruker friction and wear tester with a 4 mm GCr15 steel ball as the counterface. The test parameters included a 3 N load, a frequency of 5 Hz, a 30-minute duration, and a total stroke of 100 m. The adhesion strength of the coating was evaluated using an Rtec SMT-5000 micro and nano scratch tester under a variable loading mode, with the applied force gradually increasing from 0 to 50 N over a total scratch length of 5 mm. Each sample underwent three scratch tests to ensure accuracy, and the average values were calculated. The scratch morphologies were subsequently analyzed to assess the adhesion performance.

## Results and discussion

### Analysis of the phase composition of the MAO Coating

**Fig. 2:**
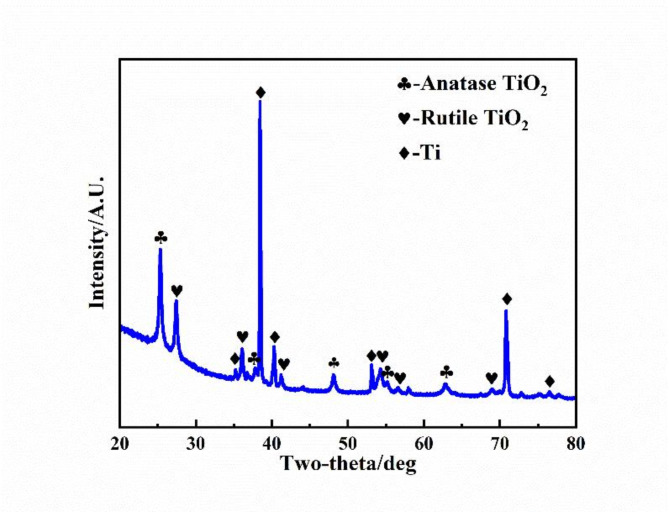
The XRD pattern of the MAO coating.

The XRD pattern of the MAO coating, as shown in Fig. 2, indicates that the primary phases present in the coating are rutile titanium dioxide (33.3% Rutile TiO₂), anatase titanium dioxide (40.2% Anatase TiO₂), and the titanium (26.5% Ti). No diffraction peaks corresponding to silicon (Si) or phosphorus (P) compounds were detected, suggesting that these elements are likely incorporated into an amorphous phase. Rutile TiO₂ and Anatase TiO₂ represent distinct crystalline forms of titanium dioxide, with Rutile TiO₂ being the thermodynamically stable phase and Anatase TiO₂ the metastable phase^22^. The diffraction peaks of Rutile TiO₂ are significantly more pronounced compared to those of Anatase TiO₂, which are relatively weak. This is consistent with reports in the literature that the Anatase TiO₂ phase transforms into Rutile TiO₂ at elevated temperatures (above 550 °C) during plasma discharge^23^. Additionally, a strong Ti diffraction peak is observed, attributed to the thin and porous nature of the MAO coating. This porous structure allows X-rays to penetrate and reach the underlying substrate, contributing to the observed intensity of the substrate diffraction peaks.

### Analysis microstructure and composite of the MAO Coating

**Fig. 3:**
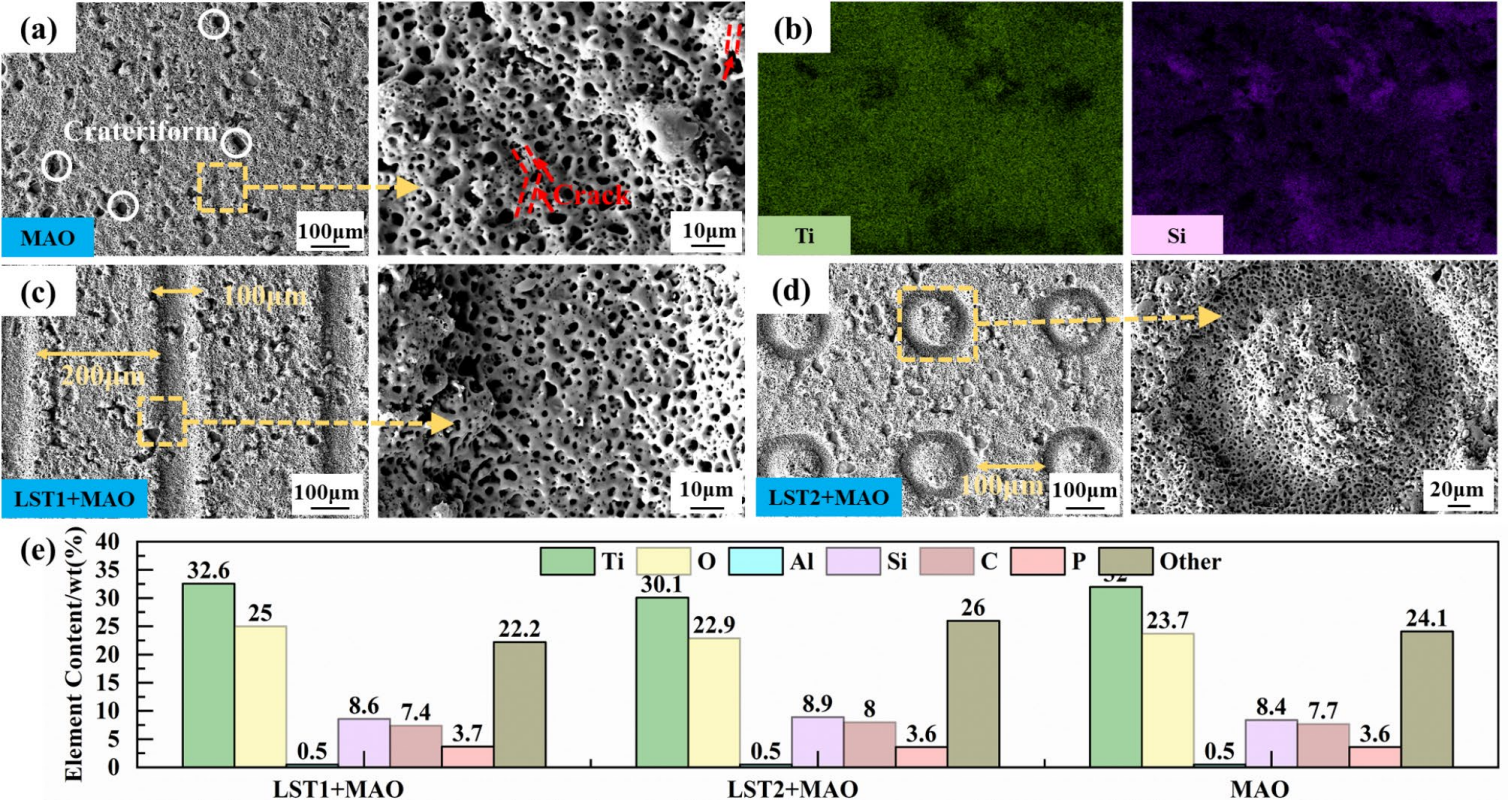
The surface morphology and composition distribution of the three types of coatings: (**a**) MAO; (**b**) EDS mapping of MAO; (**c**) LST1 + MAO; (**d**) LST2 + MAO; (**e**) EDS spectra.

Figure 3 presents SEM images of the surface morphology of three types of coating, namely, MAO, LST1 + MAO, and LST2 + MAO. Figure 3(a) shows the surface morphology of the MAO coating Without LST. After undergoing anodic oxidation, spark discharge, and micro-arc discharge in the mixed electrolyte, the surface of the TC4 substrate exhibits an uneven morphology with uniformly distributed micropores. These micropores have a volcano-like shape, formed by the ejection of molten oxides around the micro-arc discharge channels, which subsequently cool rapidly in the electrolyte and adhere to the substrate surface. Additionally, a few microcracks are observed on the coating surface, distributed in a manner that penetrates the micropores. Figure 3(c) and 3(d) display the surface morphology of the MAO coating prepared after LST1 and LST2 treatments, respectively. Both LST1 + MAO and LST2 + MAO coating exhibit an array-like distribution, with uniformly distributed pores measuring 5–6 μm in diameter. Fig. 3(b) illustrates the elemental distribution across the surface of the MAO coating, it is evident that Si is predominantly distributed within the pores of the MAO coating. Fig. 3(e) presents the statistical elemental composition of the three types of MAO coating. The data in Figure 3(e) indicate that Ti and O contents are the major constituents of the MAO coating, suggesting that the coating primarily comprises titanium oxide products. Notably, the Si content is significantly higher than that of P. This is because, during the MAO process, $\:{\text{SiO}}_{\text{3}}^{\text{2-}}$ from the electrolyte enter the discharge channels under the influence of the electric field and are directly melted, forming amorphous SiO₂. Since SiO₂ is insoluble in the alkaline electrolyte, it readily deposits on the coating surface. In contrast, $\:{\text{PO}}_{\text{4}}^{\text{3-}}$ in the electrolyte are oxidized to form P_2_O_5_, which is prone to hydrolysis, yielding species such as $\:{\text{PO}}_{\text{4}}^{\text{3-}}$, $\:{\text{HPO}}_{\text{4}}^{\text{2-}}$, or $\:{\text{H}}_{\text{2}}{\text{PO}}_{\text{4}}^{\text{-}}$. These anions undergo subsequent reactions, as reported in related literature^24–26^. Consequently, P is mainly present in the MAO coating in the form of amorphous phosphates, and P is believed to be mainly located in the amorphous phase, while the amorphous phase mainly forms the inner barrier layer, indicating that the composite electrolyte system can further improve the density and wear resistance of the coating^26,27^.

1 $$\:{\text{SiO}}_{\text{3}}^{\text{2-}}\text{+}{\text{H}}_{\text{2}}\text{O}\xrightarrow{\text{Plasma}}{\text{SiO}}_{\text{2}}\text{+}{\text{2OH}}^{\text{-}}$$

2 $$\:{\text{4PO}}_{\text{4}}^{\text{3-}}\rightarrow{\text{2P}}_{\text{2}}{\text{O}}_{\text{5}}+{\text{3O}}_{\text{2}}\uparrow+{\text{12e}}^{\text{-}}$$

3 $$\:\text{2}{\text{PO}}_{\text{3}}^{\text{-}}\text{+}{\text{H}}_{\text{2}}\text{O}\rightarrow{\text{H}}_{\text{2}}{\text{P}}_{\text{2}}{\text{O}}_{\text{7}}^{\text{2-}}$$

4 $$\:{\text{H}}_{\text{2}}{\text{P}}_{\text{2}}{\text{O}}_{\text{7}}^{\text{2-}}\text{+}{\text{H}}_{\text{2}}\text{O}\rightarrow{\text{2 H}}_{\text{2}}{\text{PO}}_{\text{4}}^{\text{-}}$$

5 $$\:{\text{H}}_{\text{2}}{\text{PO}}_{\text{4}}^{\text{-}}\rightarrow{\text{HPO}}_{\text{4}}^{\text{2-}}\text{+}{\text{H}}^{\text{+}}$$

6 $$\:{\text{HPO}}_{\text{4}}^{\text{2-}}\rightarrow{\text{PO}}_{\text{4}}^{\text{3-}}\text{+}{\text{H}}^{\text{+}}$$

7 $$\:{\text{P}}_{\text{2}}{\text{O}}_{\text{5}}\text{+}{\text{3 H}}_{\text{2}}\text{O}\rightarrow{\text{6 H}}^{\text{+}}\text{+}{\text{2PO}}_{\text{4}}^{\text{3-}}$$

**Fig. 4:**
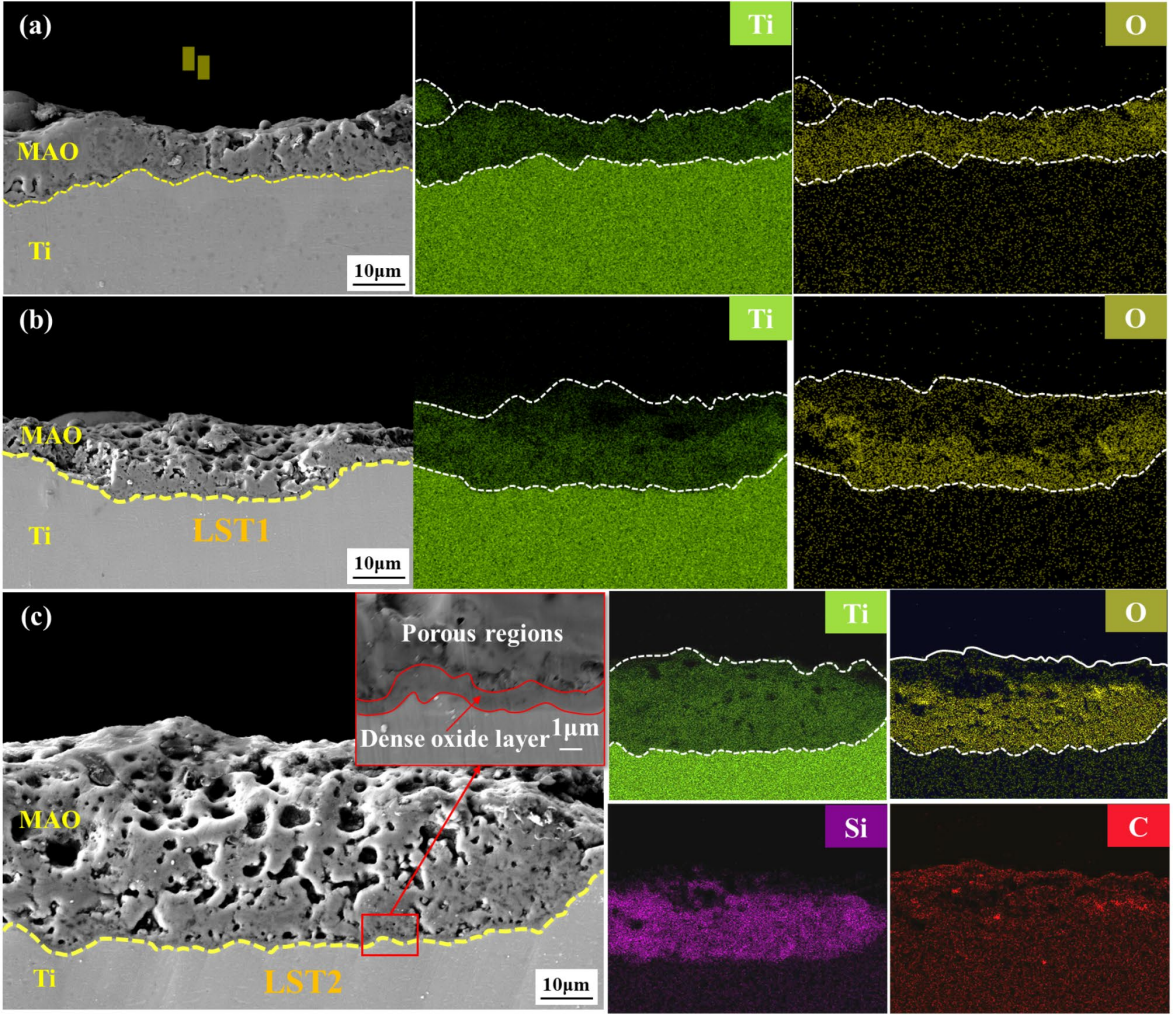
The cross-section morphology and composition distribution of the three types of coatings: (**a**) MAO; (**b**) LST1 + MAO; (**c**) LST2 + MAO.

The SEM images of the cross-sectional morphology of the three types of MAO coating are shown in Fig. 4. These images reveal that the coatings are in concave contact with the substrate, while the outer surfaces exhibit distinct protrusions. As shown in Fig. 4(a), the MAO coating without LST is relatively thin, with prominent microcracks observed at the bonding interface between the coating and the substrate. In contrast, the thicknesses of the LST1 + MAO and LST2 + MAO coating, displayed in Fig. 4(b) and Fig. 4(c), respectively, are significantly greater. This increase in thickness can be attributed to the LST treatment, which enhances surface roughness, increases the specific surface area, and raises the surface free energy of the substrate. As depicted in Fig. 4(c), the MAO coating can be divided into two distinct regions. The first is a dense oxide coating, approximately 1 μm thick, located at the interface between the MAO coating and the substrate. This coating is characterized by a compact structure with minimal porosity, containing only submicron pores, and is critical for the wear and corrosion resistance of the MAO coating. The second region is an external porous coating formed by direct deposition from the electrolyte. This porous coating contains larger discharge holes and microcracks, indicating a higher defect density. EDS analysis in Fig. 4(c) further reveals that Si content is lower in the dense oxide coating and increases at the interface between the dense and porous regions. This suggests that amorphous SiO₂, formed during the MAO process, partially fills the pores, contributing to the formation of a thin, compact coating.

**Fig. 5:**
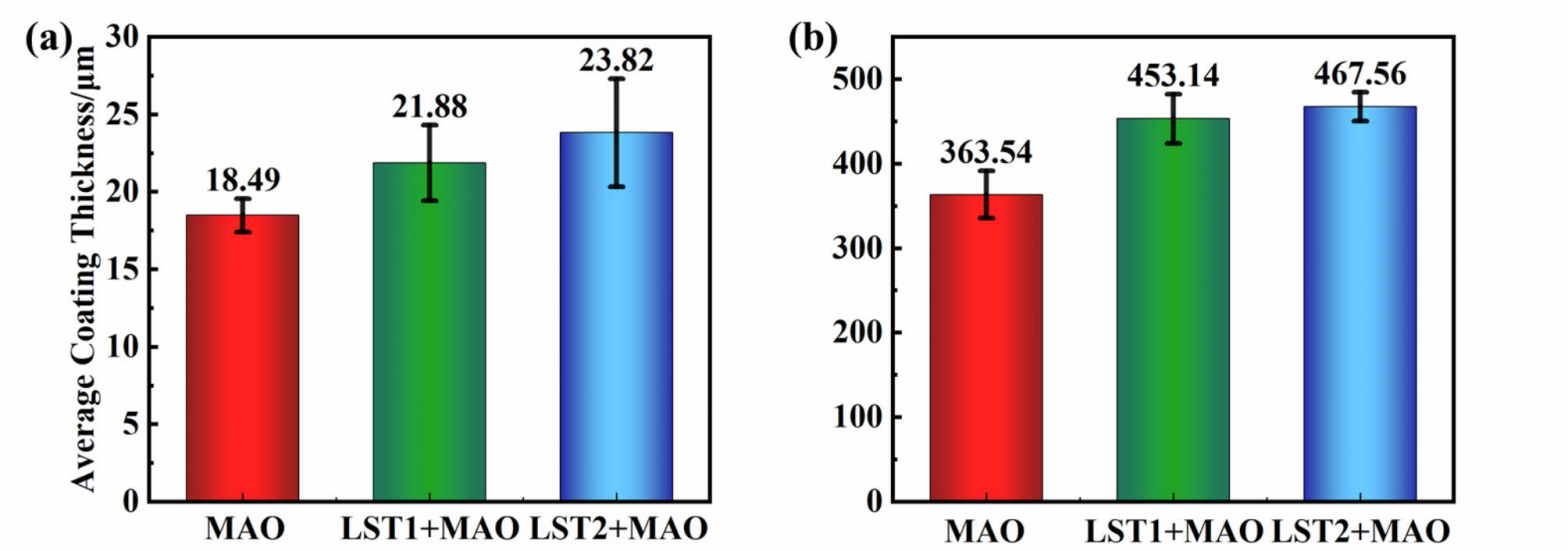
The thickness and hardness of the three types of MAO coatings: (**a**) Thickness; (**b**) Hardness.

Figure 5(a) illustrates the thickness and hardness variations of the three types of MAO coating. The coating thickness across all conditions is approximately 20 μm. The relatively higher thickness of the coating can be attributed to the substantial proportion of silicate in the electrolyte composition. Silicate ions possess a favorable oxygen atom ratio and exhibit stronger adsorption capabilities compared to phosphate ions, facilitating the growth of thicker coating. Additionally, sodium hexametaphosphate ((NaPO₃)₆) in the electrolyte promotes coating growth, enhances densification, and improves the adhesion strength between the coating and substrate^28^. Since the depth of LST2 is larger than that of LST1 and the spacing is smaller than that of LST1, its substrate-coating contact area and specific surface energy are larger, resulting in the elevated mobility of the coated ions, and the thickness of the LST2 + MAO film layer can reach 23.82 μm. Figure 5(b) shows the microhardness distribution of the three types of MAO coatings. The hardness of the laser textured MAO coating is generally higher than that of the MAO coating without LST treatment. This improvement is closely associated with the composition, thickness, and surface morphology of coating^29^. The texture treatment is carried out under the irradiation of laser. The surface of the titanium alloy is subjected to the rapid heating and cooling of the laser, the grains are refined, and the grains of the coating are preferentially oriented, making the structure denser, thereby increasing the hardness^30^. The laser textured MAO coating increases the specific surface area and surface energy of substrate, enabling the formation of thicker coatings. These thicker coatings contain higher proportions of Rutile TiO₂, contributing to increased hardness. The enhanced hardness also indirectly reflects improved adhesion strength between the substrate and the MAO coating.

**Fig. 6:**
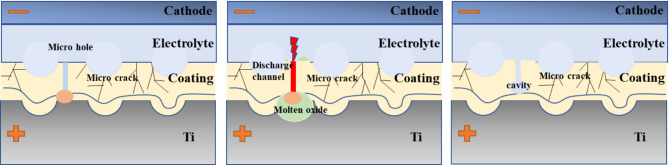
Schematic representation of the growth process for the laser-structured MAO coating.

By comparing and analyzing the microstructural morphology and properties of the three types of MAO coating, a schematic representation of the growth process for the laser-structured MAO coating was developed, as shown in Fig. 6. For the laser-structured TC4 substrate, the coating grows progressively towards the substrate during the MAO discharge process, resulting in a concave interface on the titanium substrate side and a concave-convex morphology on the coating side. As the voltage increases, dielectric instability occurs at local defects within the coating, such as cracks. These defects act as discharge initiation points, forming breakdown channels. Under the influence of a strong electric field, anions in the electrolyte penetrate these channels, moving from the outer surface to the substrate. The TC4 substrate participates in the formation of a new oxide coating, creating a dense structure at the breakdown site^31^. Conversely, regions of the coating that do not reach the substrate exhibit a looser, porous structure, highlighting the spatial heterogeneity in the coating’s growth and composition.

### Adhesion strength

**Fig. 7:**
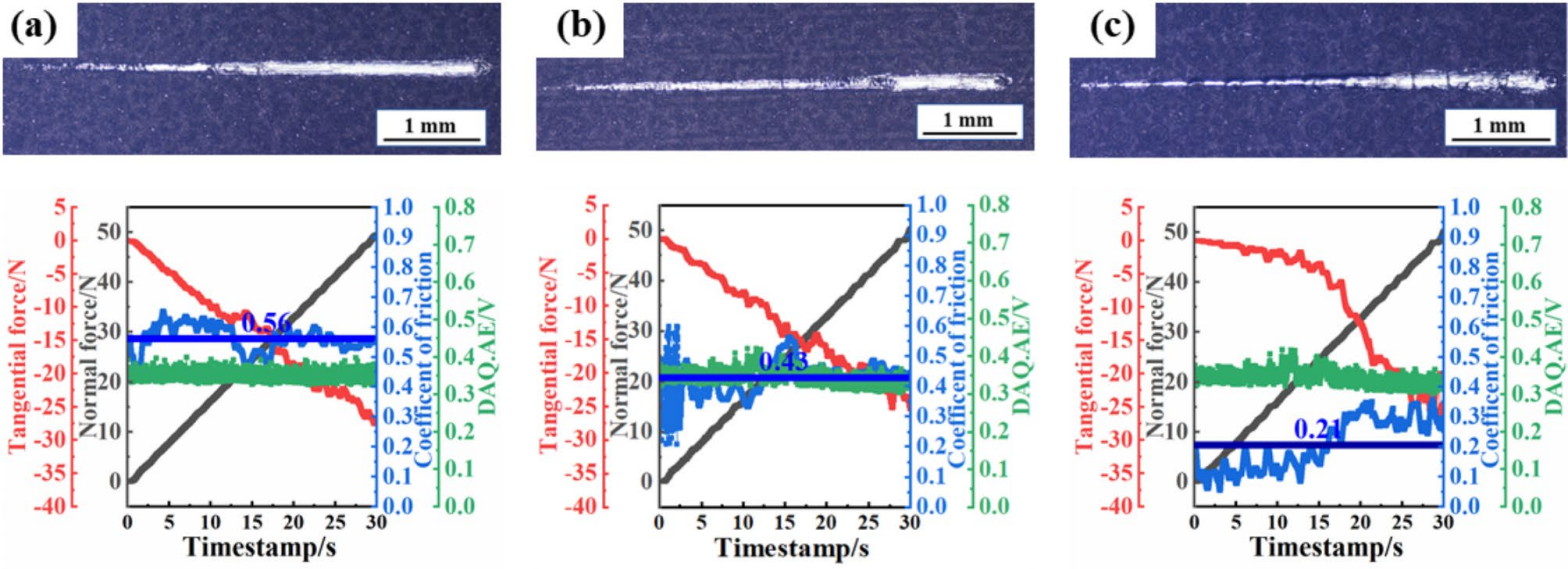
The micro-scratch morphology and scratch test results for the three types of MAO coatings: (**a**) MAO; (**b**) LST1 + MAO; (**c**) LST2 + MAO.

Figure 7 presents the micro-scratch morphology and scratch test results for the three types of MAO coating. As the applied load increases from 0 to 50 N, the scratch widths for all coating expand, accompanied by varying degrees of spalling, exposing the metallic luster of the substrate. According to tangential force method, the critical loads of MAO, LST1 + MAO and LST2 + MAO are 14.2 N, 20.9 N and 26.8 N, respectively. In Fig. 7(a), the scratches on the untextured MAO coating are the widest, with significant exposure of the metallic substrate, indicating the most severe wear. In Fig. 7(b), the scratches on the LST1 + MAO coating are narrower than those of the untextured MAO coating but still exhibit moderate spalling. In contrast, Fig. 7(c) shows that the LST2 + MAO coating exhibits the narrowest scratches and the lightest spalling. This suggests that the LST treatment increased the substrate surface roughness, thereby enhancing the bonding area between the substrate and the MAO coating. The increased roughness provided additional sites for the attachment and growth of the MAO coating^32^. This enhancement in adhesion strength mitigated the detachment and spalling of the MAO coating, improving its overall bonding performance with the substrate.

### Tribological properties of MAO Composite Coating

**Fig. 8:**
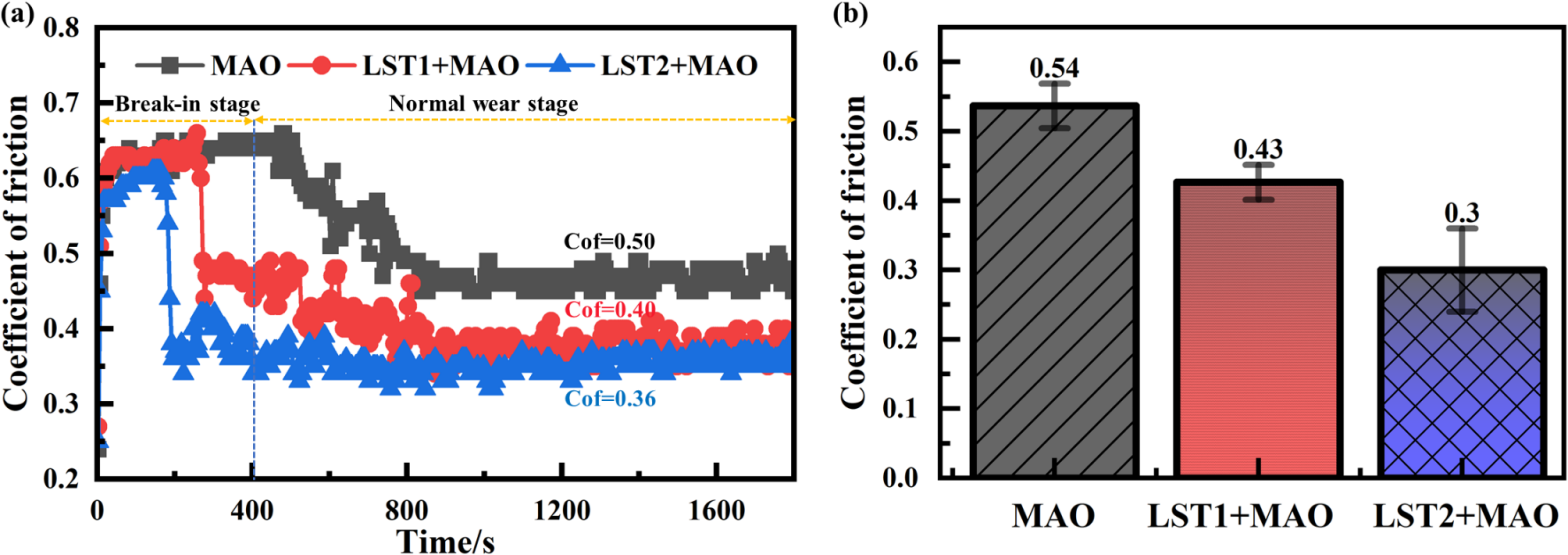
The friction coefficients of the three types of MAO coatings: (**a**) The Variation process of friction coefficients; (**b**) The average friction coefficients.

Figure 8 shows the friction coefficients of the three types of MAO coating under reciprocating friction conditions. As shown in Fig. 8(a), the friction coefficients exhibit significant fluctuations during the initial 400 s, which then gradually stabilize. These initial fluctuations are attributed to the textured treatment and the structure of the MAO coating. During the break-in stage, the loose layer of the MAO coating interacts with the counterface, leading to increased roughness and variability in the friction coefficient. In addition, as shown in Fig. 3, the surface of MAO film without laser textured treatment is smoother than that of MAO film after laser textured treatment, so its friction coefficient changes relatively slowly, and it takes a long time to enter the stable stage. As the friction process progresses, wear debris generated by the interaction gradually fills the micropores of the MAO coating and becomes compacted. This compaction reduces surface roughness, causing the friction coefficient to decrease and stabilize. In the normal wear stage, the fluctuations in the friction coefficient are gradually minimal. The untextured MAO coating stabilizes at approximately 0.5, while the LST1 + MAO and LST2 + MAO coating exhibit lower friction coefficients due to the textured treatment, which reduces wear. Specifically, the friction coefficients for the LST1 + MAO and LST2 + MAO coating stabilize around 0.40 and 0.36, respectively. Figure 8(b) summarizes the average friction coefficients, with values of 0.5, 0.43, and 0.30 for the MAO, LST1 + MAO, and LST2 + MAO coating, respectively. These results indicate that the textured treatment significantly enhances the friction reduction performance of the MAO coating, with the LST2 + MAO coating demonstrating superior friction-reducing effects compared to the LST1 + MAO coating.

**Fig. 9:**
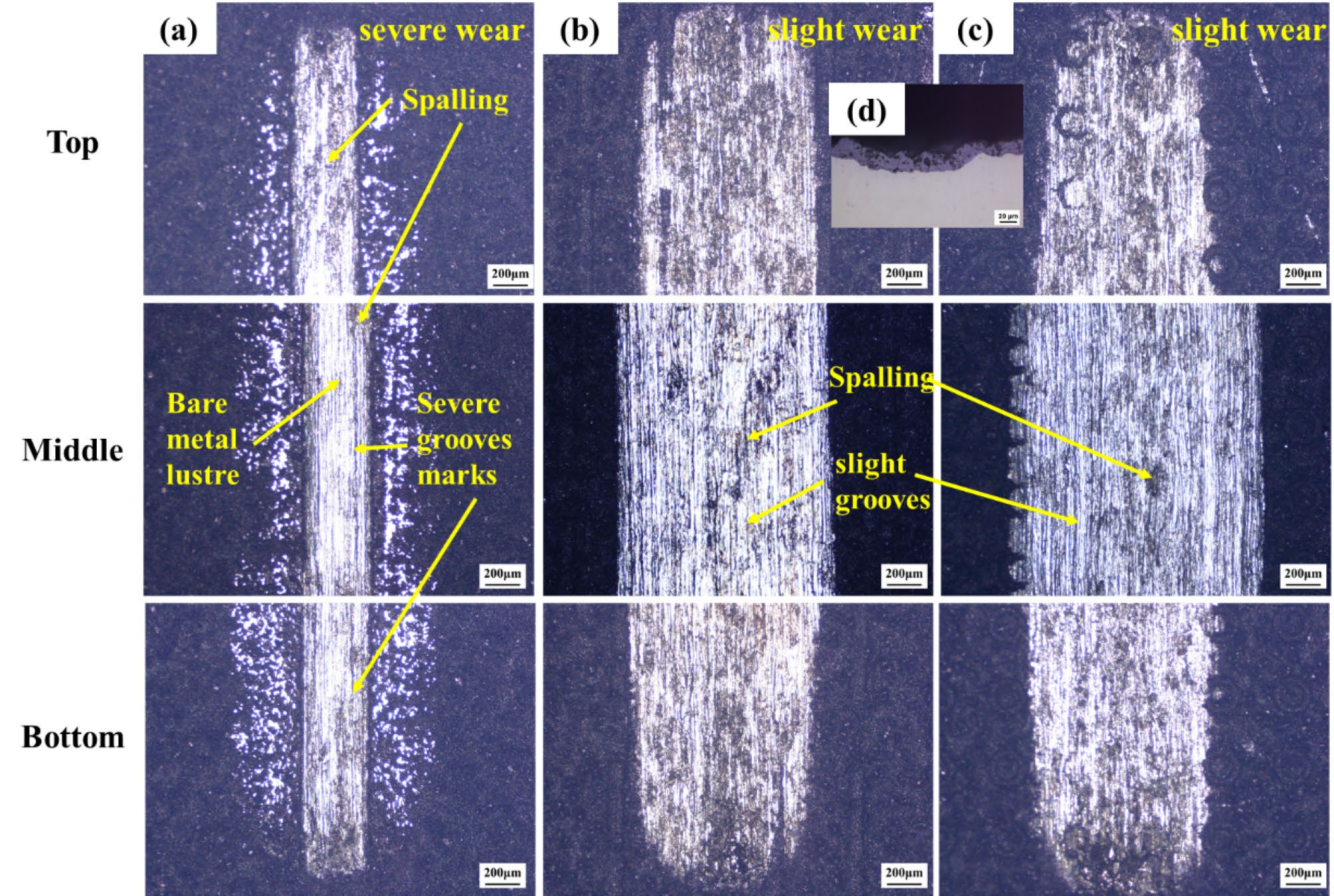
The OM images of the worn surface morphology of the three types of MAO coatings: (**a**) MAO; (**b**) LST1 + MAO; (**c**) LST2 + MAO.

Figure 9 shows the OM morphology of the wear surfaces of the three MAO film layers after reciprocating sliding friction, including the morphology of the wear surfaces at the start position, the middle position, and the end position of friction wear, with the width of the wear marks of 1.17 mm, 1.20 mm, and 1.43 mm, respectively. The narrow and deep wear marks in Fig. 9(a) illustrate that the localized stress concentration during friction leads to the coating peeling or even exposing the metal substrate, and the coefficient of friction elevated. Figure 9(b) Moderate width of wear marks but accompanied by flaking indicates that the localized coating strength is insufficient and there is uneven wear. Figure 9(c) has a large and uniform width of friction marks with shallow depth, indicating that the coating structure provides a larger area of “face contact” during friction, which disperses the contact stresses and reduces the local stress concentration. At the same time, the pores of the surface layer may have provided a lubricating effect, reducing abrasive and adhesive wear. As a result, the coefficient of friction was minimized despite the large width of the friction marks, indicating that the coating has good wear resistance and friction properties.

**Fig. 10:**
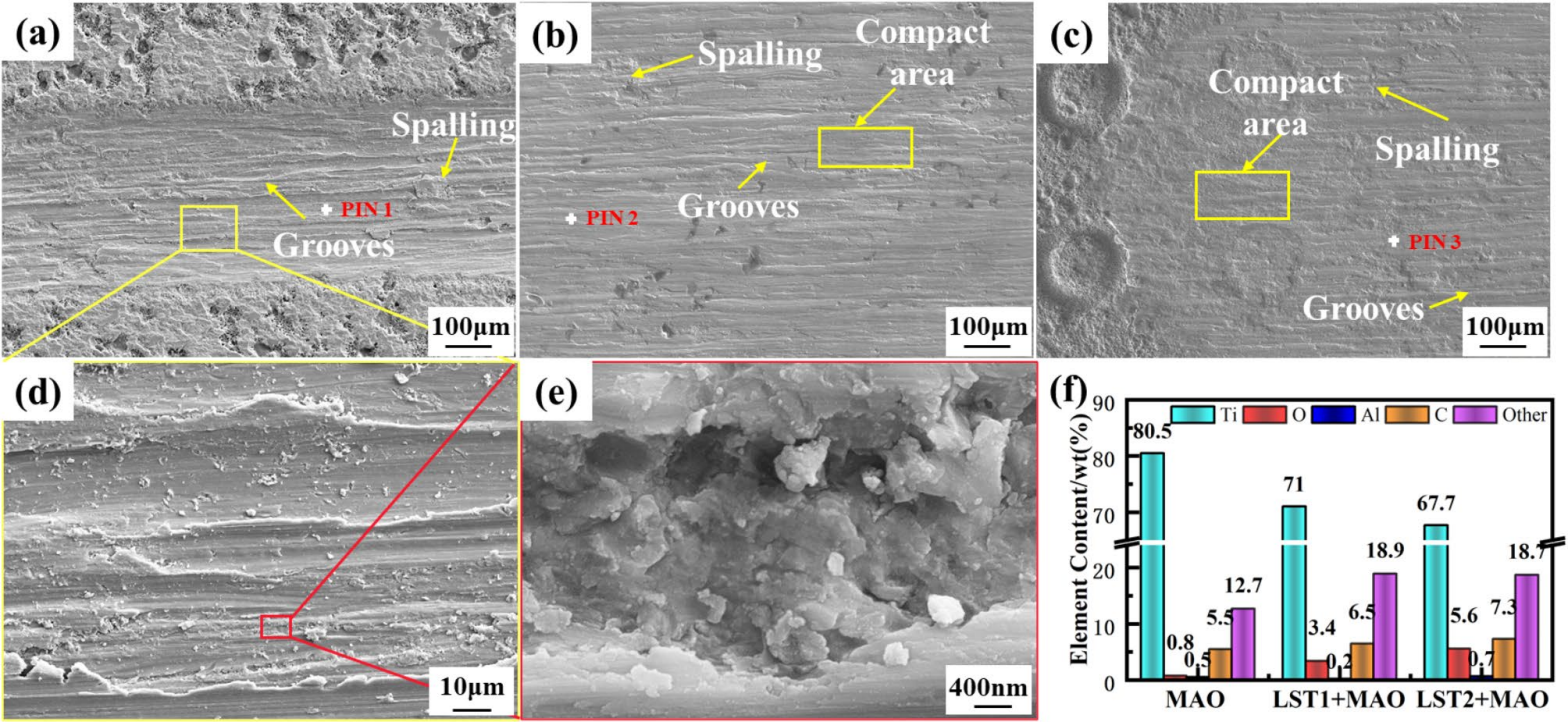
The SEM morphology and composition of the three types of MAO coatings: (**a**) MAO; (**b**) LST1 + MAO; (**c**) LST2 + MAO; (**d**) The magnified view of (a); (**e**) The magnified view of (d); (f) EDS spectra.

Figure 10 illustrates the SEM morphology and composition of the three types of MAO coating. In Fig. 10(a), the wear morphology of the untextured MAO coating is shown, exhibiting significant wear depth. The magnified view in Fig. 10(d) reveals pronounced groove features, extensive spalling pits, and adhered wear debris on the worn surface. A closer inspection, as shown in Fig. 10(e), uncovers deeper spalling pits, highlighting the severity of wear on the untextured coating. Figure 10(b-c) present the wear morphology of the LST1 + MAO and LST2 + MAO coating, respectively. The worn surfaces of these coating are comparatively smoother, with only mild groove features and small spalling craters. Notably, the characteristic LST of the MAO coating remain visible even after abrasion, indicating that the friction primarily occurred between the MAO coating and the counterface. The compositional point spectra of the worn surfaces, shown in Fig. 10(f), reveal a higher Ti content and a lower O content for all three coating. This indicates that the upper loose layer of the MAO coating has been abraded, exposing the denser underlying regions of the coating. These observations emphasize the enhanced wear resistance of the laser-textured MAO coating, with LST2 + MAO exhibiting superior tribological performance compared to LST1 + MAO and untextured MAO coating.

**Fig. 11:**
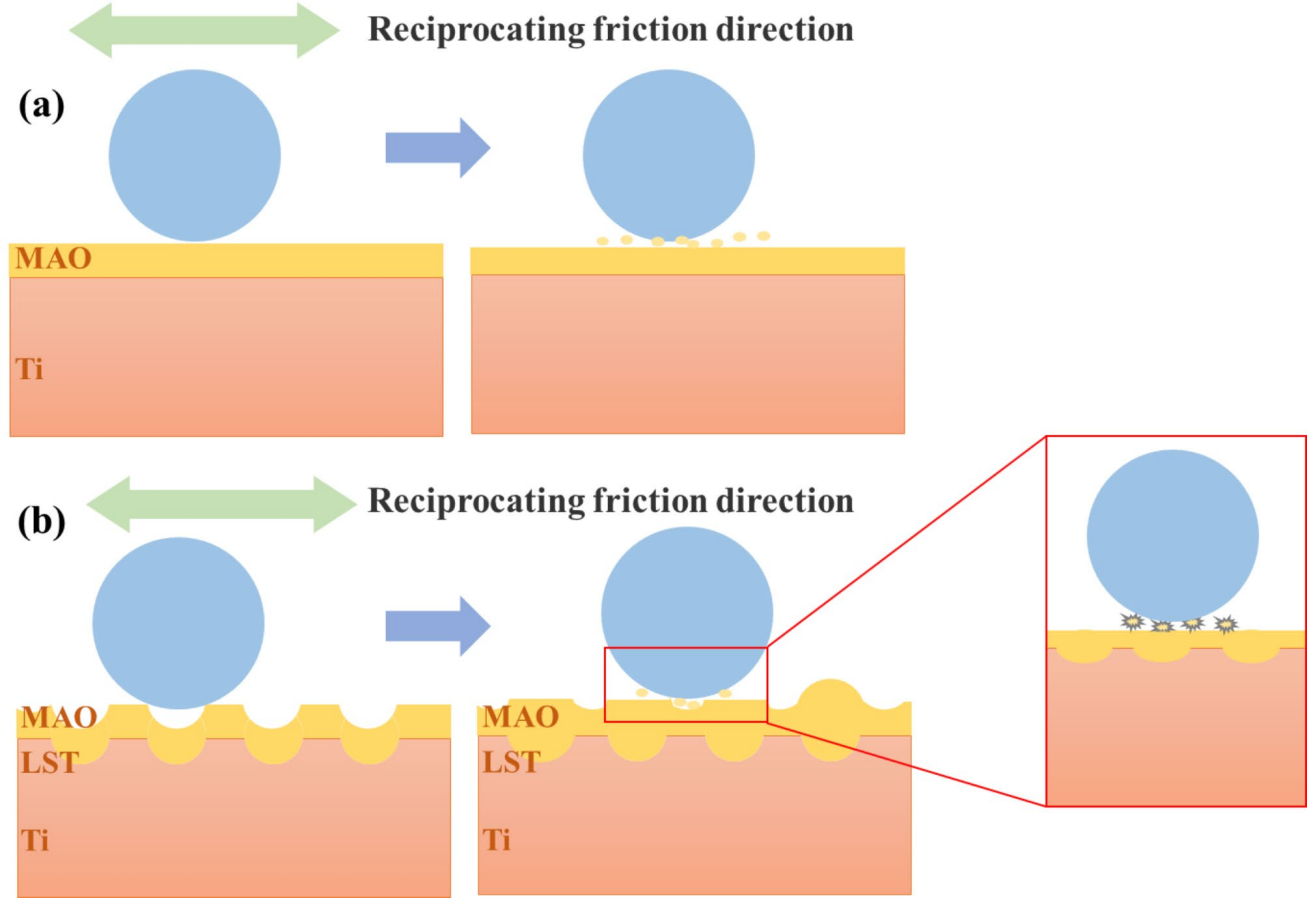
Schematic representation of the wear mechanisms of the MAO coatings: (**a**) Untextured MAO coating; (**b**)Textured MAO coating.

Figure 11 provides a schematic representation of the wear mechanisms of untextured MAO coating and textured MAO coating. In Fig. 11(a), the wear mechanism of the untextured MAO coating is illustrated. During frictional wear, the contact surface between the MAO coating and the counterface is planar. Consequently, the contact area between the counterface and the abrasive surface is relatively small. However, as reciprocating motion continues, abrasive debris is generated, with some debris becoming embedded in the micropores of the MAO coating, while the remainder acts as abrasive particles. These particles move along the counterface, leading to a severe abrasive wear mechanism across the entire worn surface. In contrast, Fig. 11(b) depicts the wear mechanism of the textured MAO coating. The uneven microstructure introduced by the LST treatment produces a non-uniform surface profile in the MAO coating. During reciprocating friction, the counterface interacts with this irregular surface, and the abrasive debris generated is partially compacted into the loose upper structure of the MAO coating. Simultaneously, some debris is trapped within the uneven microstructure. This reduces the extent of abrasive wear, resulting in a milder wear mechanism. Only small spalling pits are observed in certain areas, with slight abrasive wear and minimal spalling dominating the worn surface. This highlights the superior wear resistance of the LST + MAO coating compared to its untextured counterpart. At the same time, the existence of micro-weave structure can effectively release the residual stress of the membrane-based bonding interface to avoid the emergence and expansion of internal micro-cracks, so that the coating is not easy to peel off during friction and maintain the integrity of the coating.

## Conclusion

The LST treatment of the TC4 surface combined with a hybrid electrolyte was employed to enhance the performance of the MAO coating and elucidate its mechanisms for wear reduction and resistance. The main findings are summarized as follows:

1. The MAO coating formed on the surface of TC4 alloy consists primarily of Rutile TiO₂ and Anatase TiO₂ phases.
2. The use of a silicate-phosphate mixed electrolyte facilitated the growth of a coating with a thickness of up to 23.82 μm. The pores within the coating were uniformly distributed, with diameters of approximately 5–6 μm. The coating exhibited a dual-coating structure, consisting of a dense oxide coating (~ 1 μm) in direct contact with the substrate and a loose, porous coating on the surface.
3. The LST treatment of the TC4 surface increased the specific surface area and bonding area of substrate, thereby enhancing the adhesion strength between the MAO coating and the substrate.
4. The LST treatment reduced the average friction coefficient of the MAO coating on TC4 from 0.54 to 0.30, representing a decrease of approximately 44.4%. The wear mechanism of the untextured MAO coating was primarily abrasive wear, characterized by severe groove marks on the worn surface. In contrast, the textured MAO coating exhibited a spalling wear mechanism, with significantly less wear observed on the worn surface.

## Data Availability

The data that support the findings of this study are within the Article, or available from the corresponding author upon reasonable request.
